# Emerging role of microRNAs in diagnosis and treatment of various diseases including ovarian cancer

**DOI:** 10.1186/1757-2215-2-11

**Published:** 2009-08-27

**Authors:** Parag P Shah, Lauren E Hutchinson, Sham S Kakar

**Affiliations:** 1 Department of Physiology and Biophysics, James Graham Brown Cancer Center, University of Louisville, Louisville. KY 40202, USA

## Abstract

MicroRNAs (miRNAs) represent a class of small non-coding RNAs that control gene expression by targeting messenger RNA (mRNA). Recently, it has been demonstrated that miRNA expression is altered in many human diseases including cancers, suggesting that miRNA may play a potential role in the pathogenesis of different diseases. It has also been reported that there is a unique expression pattern of miRNAs in the disease state differing from the normal state. In this review, we focus on the miRNA signatures in different human diseases including cancers. Such signatures may be used as diagnostic and prognostic markers.

## Discovery of microRNA

Discovered in 1993 from nematode *Caenorhabditis elegans*, miRNA are small, non-coding RNAs composed of 18–24 nucleotides. They act by negatively regulating gene expression at the post-transcriptional level through modulation of mRNA expression. Lee et al. [1] found that *lin-4*, an important gene that is important for post-embryonic development of *C. elegans*, does not code for a protein. Instead, it is transcribed into a 22-nucleotide RNA molecule. Seven years later, a second miRNA, *let-7*, also emerged from *C. elegans *genetic screening. The discovery of many new miRNAs has continued there after, with evidence emerging to suggest these small RNAs play a crucial role in gene regulation. This role has been confirmed by the identification of hundreds of conserved miRNA sequences in nematodes, flies, and mammals [2].

To date, more than 3% of the genes in humans have been found to encode for miRNAs. Anywhere from 40% to 90% of the human protein encoding genes are under miRNA-mediated gene regulation [3]. MiRNAs exert their effect by negatively regulating gene expression through one of two mechanisms, i.e., mRNA degradation or translational suppression. The mechanism by which the miRNA proceeds is dependent on the complementarity between the miRNA and its mRNA target [4]. A high level of complementarity corresponds to the degradation of the target mRNA via the RNA-mediated interference (RNAi) pathway. When there is inadequate complementarity, miRNAs bind to the untranslated region of the target mRNAs and translational suppression occurs through a RNA-induced silencing complex (RISC). Plants more commonly utilize the first mechanism while animals employ the second [2].

MiRNAs play an important role in different biological process such as cell proliferation during development, cell growth, and organization as well as apoptosis, biogenesis, transcription, signal transduction, cell cycle, neurogenesis, and fat metabolism. The deregulation of miRNA function can lead to human diseases as the target genes of miRNA are involved in several metabolic disorders such as various forms of cancers, lymphomas, diabetes and neurological disorders such as Parkinson's and Alzheimer's diseases. Recently, there has been considerable interest in understanding the mechanism of the RNA regulatory phenomena and how miRNA functions in carcinogenesis. Specifically, determining how the regulation of miRNA may be targeted to develop cancer therapies has been a focus. In this review, we discuss recent discoveries related to miRNA in different cancer diagnoses and, in particular, treatment related to ovarian cancer (OVCA).

## Biogenesis of miRNA

**Figure 1:**
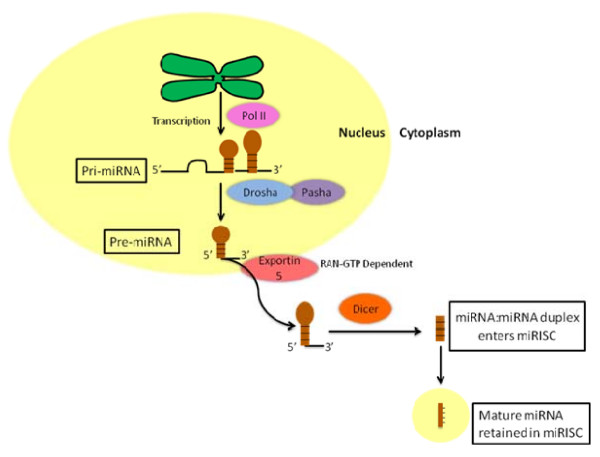
**Systematic miRNA biosysnthesis and trafficking to cytoplasm**.

Like proteins, miRNAs are transcribed from DNA into a primary (Pri) RNA transcript. In the first step, formation of Pri-miRNA inside the nucleus occurs from the transcription of the miRNA gene by RNA polII. The Pri-miRNA is capped, polyadenylated, and then processed by RNAase III endonuclease Drosha and the double stranded (ds) RNA-binding protein Pasha. This processing results in the formation of a 60 to 70 nucleotide stem loop precursor (Pre) called Pre-miRNA. The Pre-miRNA is then transported to the cytoplasm by the Ras-related nuclear protein-guanosine-5'-triphosphate (RAN-GTP)-dependent export receptor Exportin-5. Next, the Pre-miRNA is cleaved at the base of the loop by a second RNase III endonuclease, a cytoplasmic Dicer [5]. This forms a transient, double stranded miRNA (dsmiRNA) of 22 nucleotides in length. The dsmiRNA complex then enters the miRNA-associated multiprotein RISC (miRISC), which contains the Argonuate 2 protein, and the mature miRNA is retained in the miRISC complex [2]. Argonuate proteins are active RNase enzymes and extremely conserved in the RISC. The mature miRNA is now capable of regulating its target mRNAs (See Fig. 1). Deregulation of this processing results in perturbation in miRNA genesis and may have oncogenic consequences.

## Role of miRNAs in disease

Gene regulation by miRNA can affect a wide variety of cell functions, such as regulation of cell differentiation, proliferation, and apoptosis. MiRNAs are important for normal cell functioning; dysfunction of the miRNA regulation system results in disruption of the normal cellular activity leading to induction of a disease.

### MiRNAs in neurodegenerative diseases

Neurodegenerative diseases are the result of the deterioration of neurons, which leads to disabilities and the possibility of death. Studies have shown that gene defects play an important role in the pathogenesis of neurodegenerative diseases such as Parkinson's, Alzheimer's, and Huntington's [6].

Parkinson's disease is the second most prevalent age-associated neurodegenerative disorder. Like Alzheimer's disease, gene mutations can result in inherited forms of Parkinson's disease [7]. Kim et al. [8] suggest that miRNAs are essential for maintaining the dopaminergic neurons in the brain. Given that Parkinson's disease is characterized by an extreme loss of dopaminergic neurons, it could be possible that miRNAs play a role in the disease pathogenesis. These investigators [8] hypothesized that inducing alterations in the miRNA networks of the brain results in neurodegenerative disorders to some extent. Based on the results of this study and from information obtained in previous work performed with mice [9], flies [10] and cultured neurons [8] in which the enzyme Dicer was genetically inactivated, Kim et al. [8] concluded that loss of Dicer results in complete inactivation of miRNA and is tremendously detrimental [11]. Moreover, Dicer was shown to be important for neuronal survival, as the loss of miRNAs may result in development and/or progression of Parkinson's disease [8,9].

Spinal muscular atrophy (SMA) is a common genetic disease characterized by the progressive degeneration of motor neurons. It has been demonstrated that deletion or loss-of-function mutations in the survival of motor neuron (SMN) protein results in SMA [12]. Gemin 3 and Gemin 4 are shared components of SMN and miRNA-containing ribonucleoprotein particles (miRNPs). Deletion or loss-of-function mutations of SMN in SMA may affect the activity of miRNPs due to possible redistribution or changes in the levels of Gemin 3 and 4.

Using RNA immunoprecipitation 3'-end labeling and miRNA cloning, Dostie and colleagues [13] identified 53 novel miRNAs from mouse and human cell lines, out of which several were found to be phylogenetically conserved. Additionally, several miRNAs that were discovered to be components of miRNPs in these cells seem to represent distinct subfamilies, all of which contain multiple copies of these miRNAs on different chromosomes. As such, it can be proposed that these miRNAs function to regulate gene expression.

Julie Bilen and colleagues [14] isolated miRNA bantam (ban) in the genetic screen for the modulators of pathogenecity of the human neurodegenerative disease model in Drosophila. They demonstrated that upregulation of ban mitigated degeneration induced by the pathogenic polyglutamine (polyQ) protein Ataxin 3, which was mutated in human polyQ disease spinocerebellar ataxia type 3 (SCA 3). They also showed that loss of all miRNAs by Dicer mutation dramatically enhances pathogenic polyQ protein toxicity in flies and human HeLa cells. These studies suggest that miRNAs may be important for neuronal survival in relation to neurodegenerative disease.

### MiRNAs in heart diseases

The heart responds to chronic and acute injury by hypertrophic growth. Cardiomyocyte hypertrophy is the dominant cellular response to virtually all forms of hemodynamic overload, endocrine disorders, myocardial injury, or inherited mutations in the various structural and contractile proteins [15]. Multiple genes are aberrantly expressed in hypertrophic cardiac cells. Using microarray analysis, miRNA expression pattern in hearts revealed that miR-1, miR-29, miR-30, miR-133, and miR-150 have often been found to be down-regulated while miR-21, miR-125, miR-195, miR-199, and miR-214 are up-regulated with hypertrophy.

MiR-21 is found to be consistently induced by cardiac stress and appears to function as a regulator of cardiac growth and fetal gene activation in primary cardiomyocytes *in vitro*. Its role in myocyte hypertrophy was demonstrated by Cheng et al. in 2007 [16]. Their study showed that knockout of miR-21 by an inhibitor suppresses cardiomyocyte hypertrophy induced by angiotensin II in cell culture experiments [16]. This indicates that overexpression of miR-21 has a functional role in cardiac hypertrophy. Tatsuguchi et al. [17] also confirmed the role of miR-21 in cardiac hypertrophy using locked nucleic acid modified oligos. Recently, Sayed et al. [18] reported that miR-21 modulates the cellular outgrowths during hypertrophy through the regulation of sprouty 2, an intracellular inhibitor of mitogen-activated protein-kinase signaling.

Another miRNA that is consistently up regulated in rodent and human hypertrophic hearts is miR-195. Forced expression of miRNA-195 in transgenic mice is sufficient to induce hypertrophic growth and also results in dilated cardiomyopathy and heart failure [19].

MiR-208 is encoded within intron 27 of the α major histocompatibility complex (MHC) gene and plays a key role in the expression of β MHC in response to cardiac stress [20]. Although the expression level of miR-208 remains stable during cardiac stress, this miRNA appears to fulfill a dominant function in regulating cardiac hypertrophy and remodeling. In response to pressure overload by thoracic aortic constriction, the knock-out mice show virtually no hypertrophy of cardiomyocytes or fibrosis and are unable to up regulate the β MHC expression [19]. MiR-208 is supposed to act by repressing the expression of a common component of stress-response and thyroid hormone signaling pathways in the heart, that of the thyroid-hormone-receptor (TR) co-regulator TR-associated protein 1 (THRAP1).

MiR-1 is a muscle-specific miRNA that forms a bicistronic cluster with miR-133. Both miRNAs belong to the same transcriptional unit and have been found to play a crucial role in determining when cardiomyocyte hypertrophy occurs [21]. While both miRNAs are expressed at low levels in the mouse and human models of cardiac hypertrophy, their overexpression can inhibit cardiac hypertrophy.

### MiRNAs in diabetes

Diabetes is a chronic disease caused by defects in insulin production, secretion, and signaling [22]. Poy et al. [12] reported that overexpression of miR-375 resulted in suppressed glucose-stimulated insulin secretion and its inhibition resulted in enhanced insulin secretion. Myotrophin, a protein that has a role in exocytosis, was identified as one of the targets of miR-375. Inhibition of Myotrophin production has similar effects of miR-375 on insulin secretion, making miR-375 an important modulator of β-cell function. Keller et al. [23] suggested the additional targets of miR-375 could be the pancreatic transcription factors pdx-1 and NeuroD1. This indicates that miR-375 may play important role in interacting with transcription factors that control pancreatic maintenance and development [24].

## MiRNA and carcinogenesis

Recent studies show that several miRNAs are differentially expressed in several human cancers, which indicate that miRNAs may have a role in the carcinogenic processes (See Table 1). In 2002, Calin et al. [25] correlated aberrant miRNA expression with cancer. The first study that was reported regarding the abnormalities in miRNA expression was in miR-15a and miR-16-1 in B-cell chronic lymphocytic leukemia (CLL); [25]. The study showed that miRNA 15 and miRNA 16 genes are located in a deleted region present in more than half of B-cell CLL cases and that both of the genes are deleted/mutated in more than half of the in CLL cases. In addition, miR-17-92, which is present as a cluster of miRNAs on chromosome 13, was reported to be associated with B cell lymphoma [26]. Other miRNAs that have been discovered to be abnormally expressed in cancerous tissues include: miR-145, which is down regulated in breast cancer [27] ovarian cancer [4]; miR-10b, which is found to be mostly down regulated in breast cancer [27]; and miR-145, which has reduced expression in colorectal cancer [28].

**Table 1:** Differentially expressed miRNAs in various cancers.

miRNAs	Cancer types	Reference
mir-17-92 cluster	β-cell lymphoma lung carcinoma	Hayashita et al. (2005) [36]
	lung carcinoma	Hayashita et al. (2005) [36]
		
miR-145	colon, breast, lung and Prostate	Cummins et al. (2006) [47]
		Akao et al. (2006) [48]
		Bandres et al. (2006) [49]
		Iorio et al. (2005) [27]
		Michael et al. (2003) [28]
		Yanaihara et al. (2006) [50]
		
miR-15a and miR-16-1	chronic lymphocytic leukemia (CLL)	Calin et al. (2005) [51]
		
miR-221	papillary thyroid carcinoma	He et al. (2005b) [52]
		
miR-21	glioblastoma	Chan et al. (2005) [53]
		
miR-122a	Hepatocellular carcinoma	Kutay et al. (2006) [54]
miR-125a, miR-139		Gramantieri et al. (2007) [55]
miR-150, miR-145		Meng et al. (2007) [56]
		Jiang et al. (2008) [57]
		
miR-205	Ovary, Lung, Bladder, Head and Neck	Ioria et al. (2007) [4]
		Yanaihara et al. (2006) [50]
		Gottardo et al. (2007) [58]
		Tran et al. (2007) [59]
		
miR-183	Bladder, Colonrectal	Gottardo et al. (2007) [58]
		Bandres et al. (2006) [49]
		
miR-95	Cervix	Cheng et al. (2005) [60]
		
miR-155	Breast, Lung, Pancreas, CLL	Iorio et al. (2005) [27]
		Yanaihara et al. (2006) [50]
		Gironella et al. (2007) [42]
		Lee et al. (2007) [61]
		Fulci et al. (2007) [62]
		
miR-200a	Ovary	Iorio et al. (2007) [4]
		
miR-301	Pancreas	Gironella et al. (2007) [42]
		Lee et al. (2007) [61]
		
miR-141	Ovary	Iorio et al. (2007) [4]
		
miR-106a	Endomertium,	Boren et al. (2008) [63]
	Neuroblastoma,	Schulte et al. (2008) [64]
	T-cell leukemia	Landais et al. (2007) [65]
		
miR-17-5p	Bladder and Lung	Gottardo et al. (2007) [58]
		Matsubara et al. (2007) [66]
		
miR-34a	Ovary, Lymphocytic	Iorio et al. (2007) [4]
	leukemia	Zanette et al. (2007) [67]
		
miR-200c	Ovary	Iorio et al. (2007) [4]
		
miR-23a	Lung	Yanaihara et al. (2006) [50]

MiRNAs participate in various biological processes including cell differentiation, proliferation, apoptosis, stress resistance, and fat metabolism [29] and often have dual functions. For instance, in some types of cancers, they possess oncogenic activity, while in others they can suppress tumor development. Michael et al. [28] showed that miR-143 and mi-R-145 have been associated with the reduction of some malignancies, suggesting their potential tumor suppressing activities. Additional studies demonstrated that miRNAs were found to suppress BCL2, an anti-apoptotic gene [30]; supporting the idea that miRNA can also have oncogenic activity. Furthermore, miR-372 and miR-373 cooperate with oncogenic Ras in the cellular transformation of testicular germ cell tumors [31]. Additionally, in the Let-7 family, Let 7a-2 and Let 7a-3 are down regulated in lung cancer and Ras is frequently mutated [32]. Let-7b is involved in Ras regulation and inhibits cell cycle progression in melanoma [33,34].

MiR-17-92 has been shown to function in embryogenesis [35] and is overexpressed in lung cancer, especially in small cell lung cancer, which is characterized by neuroendocrine differentiation. In addition to being overexpressed, there are an increased number of gene copies. This suggests they have a role in the development of lung cancer [36]. MiR-34a, which is absent in pancreatic cells, was directly trans-activated by the p53 gene and proved to be associated with modulation of gene expression initiated by p53 [37]. C-MYC, a proto-oncogene, encodes for a transcription factor that is responsible for cell proliferation and growth. A mutation in this gene results in most common abnormalities associated with human cancers. Finally, using microarray-based large scale profiling, Volinia et al. [38] analyzed the miRNA expression pattern in 540 normal and tumor samples from six types of tissues (lung, breast, stomach, prostate, colon, and pancreas). They proved that the abnormal expression pattern of many miRNAs is connected to carcinogenesis.

For additional information regarding differential expression of miRNAs in various cancers including glioblastomas, colorectal neoplasia, hepatocellular carcinoma, papillary thyroid carcinoma etc. ' [see Additional file 1]'.

## MiRNAs in human ovarian carcinoma (OVCA)

OVCA is the most common of the gynecologic malignancies, but little is known about the molecular genetics of its initiation and progression. Epithelial OVCA (EOC), which accounts for approximately 90% of OVCAs, continues to be the leading cause of death resulting from gynecological malignancies [39]. Histologically, OVCA is divided into different subgroups such as serous, mucinous, endometrioid, clear cell, Brenner, and undifferentiated carcinomas [40]. The high mortality rate of this disease is due to late stage diagnosis for more than 70% of affected patients (Chemoresistance). Trans-vaginal ultrasound and serum CA-125 remain poor tests for diagnosing the disease in its early stage and, as a result, most cases are still more likely to be detected at the advanced stage [41].

New, more effective diagnostic tests must be developed to identify OVCA in its early stage. If OVCA is diagnosed in the early stage, current treatments will be more successful, which in turn will significantly reduce the high mortality rate. The discovery that miRNAs are expressed at different levels in normal versus malignant tissues offers the possibility of identifying new methods of diagnosing early stage incidence and discovering new therapies for the treatment of OVCA.

With the advent of new miRNA technology, it is possible to plan appropriate treatments and predict chemotherapy outcomes. Studies have shown that miRNAs, which are noncoding regulatory RNA products, are aberrantly expressed in many cancer types including breast [27], pancreatic [42], and lung cancer [4]. In addition to these forms, a study conducted by Iorio et al. in 2007, [4] confirmed that miRNAs are also abnormally expressed in OVCA (See Table 2). It was concluded that in the cancerous tissues, i.e., miR-141, miR-200a, miR-200b, and miR-200c, had the highest level of up-regulation while miR-125b, miR-140, miR-145, and miR-199a were the most down-regulated. Moreover, this study demonstrated that the levels of expression between the normal and malignant ovaries were so drastic that expression levels alone could distinctly distinguish between tissue types. This finding is extremely significant because it points to the possibility that miRNAs may have oncogenic or tumor suppressing activities.

**Table 2:** Differentially expressed miRNAs in ovarian cancer.

miRNAs	Up/Down-regulated	Reference
miR-21, miR-92, miR-93, miR-126, miR-29a	up-regulated	Resnick et al., 2009[43]
		
miR-155, miR-127, miR-99b	down-regulated	Resnick et al., 2009[43]
		
miR-200a, miR-141, miR-200c, miR-200b	up-regulated	Iorio et al., 2007[4]
		Zhang et al., 2006 [39]
		
miR-199a, miR-140, miR-145, miR-125b1	down-regulated	Iorio et al., 2007 [4]
		Zhang et al., 2006 [39]
		
miR-100, miR-199a, miR-295, miR-29a, miR-29c, miR-99a, miR-494	up-regulated	Dahiya et al., 2008 [68]
		Kerscher et al., 2006 [2]
		
let-7d, miR-106b, miR-122a, miR-141, miR-183, miR-195, miR-200a, miR-335, miR-424, miR-155, miR-488, let-7a	down-regulated	Dahiya et al., 2008 [68]
		Iorio et al., 2007 [4]
		Zhang et al., 2006 [39]

In addition to the difference in expression levels between normal and cancerous tissues, it has been shown that miRNAs can also be histotype-specific. In endometrioid tumors, miR-21, miR-182, and miR-205 are considerably overexpressed while miR-144, miR-222, and miR-302a have reduced expression compared to normal. However, in serous and clear cell tumors, these miRNAs are normally expressed [4]. These findings suggest that while all types of OVCA are currently treated with the same standard therapies, a more effective course of treatment could be targeted towards specific histotypes of various cancers.

The above tests are invasive procedures because they require a tissue sample to determine the expression levels of miRNAs for diagnostic purposes. However, a new non-invasive approach has recently been discovered that only requires a serum sample from the patient. In the serum of OVCA patients, miRNAs 21, 29a, 92, 93, and 126 were overexpressed while miRNAs 127, 155, and 99b were under expressed as compared to controls. This indicates that a patient's serum miRNA level could be used to potentially diagnose OVCA. Additionally, miRNAs 21, 92, and 93 were also overexpressed in patients with normal CA125 levels. This suggests that earlier diagnosis of OVCA may be possible through the identification of these miRNAs even though patients may present with normal CA-125 levels [43].

Another new non-invasive method with the potential for diagnosing OVCA involves isolating tumor-derived exosomes. Exosomes are saucer shaped nanovesicles (30–100 nm) that are normally released by multiple mammalian cell types and have a significant role in cell-to-cell communication. In addition to exosomes released by normal proliferating cells, tumor cells also release exosomes. Tumor cells release exosomes at a much higher level than normal cells and the exosomes also display numerous tumor antigens that mirror the primary tumor cells. Therefore, it is proposed that these tumor-derived exosomes could be used as a diagnostic biomarker. In addition to the antigens, circulating tumor-derived exosomes have been shown to contain miRNAs that parallel those of the original tumor [44]. This demonstrates that miRNA profiling can be executed and accurately represent the originating tumor through a blood sample rather than requiring the more invasive tissue sample. It has been shown that eight particular miRNAs are of diagnostic significance in OVCA. MiRNA 21, 141, 200a, 200b, 200c, 203, 205, and 214 are all overexpressed in tumor cells and tumor-derived exosomes. The levels are different enough to clearly distinguish between benign and malignant tumors as well [44].

The current treatment utilized for patients with OVCA consists of primary cytoreductive surgery followed by platinum and taxane combination chemotherapy treatments. This method of therapy is initially successful; however, most of the patients become resistant to the platinum-based chemotherapy [45]. Recent studies indicate that there are significant differences in the level of miRNA expression between chemotherapeutic sensitive and resistant OVCA cell lines and tissues. Boren et al. [45] found that 27 miRNAs were connected to OVCA cell line sensitivity to platinum-based chemotherapeutic agents and the differential expression of these miRNAs leads to chemoresistance. In the cells resistant to the drugs, 18 miRNAs were up-regulated and 9 were down-regulated. It was determined that increased expression of miR-181a, miR-181b, and miR-213 in OVCA cell lines results in resistance to doxorubicin and gemcitabine and increased expression of miR-99b and miR-14 leads to docetaxel and paclitaxel resistance. Eitan et al. [46] also discovered several miRNAs that were differentially expressed in stage 3 ovarian tumors. It was established that increased expression of miR-23a, miR-27a, miR-30c, let-7g, and miR-199a-3p corresponds to resistance to platinum-based chemotherapy while reduced expression of miR-378 and miR-625 relates to resistance.

The difference in miRNA expression levels between chemotherapy-sensitive and resistant cells is clinically significant. New treatments could exploit these differences if the mechanism behind regulating miRNA expression is determined. Then, for instance, miR-378, which is down-regulated in malignant ovarian tissue, could be induced to over express to a normal level, possibly making the cells more sensitive to platinum-based chemotherapy. Moreover, identification of specific miRNAs between types of cancers (lung, ovarian, pancreatic) and even within types (endometrioid, clear cell, serious in ovarian) could allow for more personalized treatments, which certainly present the chance for a better prognosis.

While there is not much information currently available on the mechanisms responsible for inducing aberrant miRNA expression, there is a new possible explanation for their overexpression in OVCA. Iorio et al. [4] recently proved that treating OVCAR3 cells with 5-aza-2' deoxycytidine, a demethylating treatment, results in the overexpression of miR-21, miR-203, and miR-205. These miRNAs are also up-regulated in ovarian malignant tumors. This indicates that the mechanism causing their up-regulation *in vivo *could be DNA hypomethylation.

## Conclusion

The discovery of aberrant miRNA expression between control patients and those with OVCA holds great promise for improved patient care and more positive outcomes. Thus, identification of altered miRNA expression, as well as their targets, provides new opportunities for therapeutic strategies. MiRNA-based gene therapy targeting deregulated miRNAs will be the future tool for cancer treatment. Using data from miRNA profiling and knowledge of familial history of a certain diseases will help in developing miRNA-based personalized therapy. Detailed understanding of the characteristic miRNA abnormalities could contribute to novel approaches in early diagnosis and treatment of different diseases including cancer and can serve as a biomarker for cancer. miRNA expression profiling eventually assist physicians in providing patients with accurate diagnosis, personalized therapy, and treatment outcome.

## Abbreviations

(miRNA): MicroRNA; (mRNA): messenger RNA; (RNAi): RNA-mediated interference; (RISC): RNA-induced silencing complex; (Pri): primary; (Pre): precursor; (RAN): Ras-related nuclear protein; (GTP): guanosine-5'-triphosphate; (ds): double stranded; (dsmiRNA): double stranded miRNA; (miRISC): miRNA-associated multiprotein RISC; (SMA): Spinal muscularatrophy; (SMN): survival of motor neuron; (miRNP): miRNA-containingribonucleoprotein particle; (ban): bantam; (polyQ): polyglutamine; (SCA 3): spinocerebellar ataxia type 3; (MHC): major histocompatibility complex; (TR): thyroid-hormone-receptor; (THRAP1): thyroid-hormone-receptor co-regulator TR-associated protein 1; (CLL): chronic lymphocytic leukemia; (EOC): Epithelial ovarian cancer; (OVCA): ovarian cancer.

## Competing interests

The authors declare that they have no competing interests.

## Authors' contributions

PPS drafted the manuscript. LEH contributed in writing part of the manuscript. SSK conceptualized, edited, and revised the manuscript. All authors have read and approved the final manuscript.

## Supplementary Material

Additional file 1 **Differentially expressed miRNAs in various diseases**. The data provided represents the differential expression of miRNAs in various diseases. Also includes cited references for this file. Click here for file
