# Supplementary material for: Emerging role of microRNAs in diagnosis and treatment of various diseases including ovarian cancer
Source: J Ovarian Res. 2009 Aug 27;2:11. doi: 10.1186/1757-2215-2-11 (PMC2744658; doi:10.1186/1757-2215-2-11)
Supplement: Additional file 1 — Differentially expressed miRNAs in various diseases. The data provided represents the differential expression of miRNAs in various diseases. Also includes cited references for this file. [file 1757-2215-2-11-S1.doc]

**Additional file 1. Differentially expressed miRNAs in various diseases.**

miRNAs Disease types Up/Down-regulated Reference

let-7a, let-7b, let-7c, prostate cancer down-regulated Porkka et al., 2007 [1]

let-7d, let-7g, miR-16,

miR-23a, miR-23b,

miR-26a, miR-92,

miR-99a, miR-103,

miR-125a, miR-125b,

miR-143, miR-145,

miR-195, miR-199a,

miR-199a, miR-221

miR-222, miR-497

miR-202, miR-210, prostate cancer up-regulated Porkka et al., 2007 [1]

miR-296, miR-320,

miR-370, miR-373,

miR-498, miR-503

miR-16, miR-92a, prostate cancer up-regulated Lodes et al., 2009 [2]

miR-103, miR-107,

miR-197, miR-34b,

miR-328, miR-485-3p,

miR-486-5p, miR-92b,

miR-574-3p, miR-636,

miR-640, miR-766,

miR-885-5p

miR-21, miR-155, pancreatic cancer up-regulated Bloomston et al., 2007

miR-221 [3]

miR-155, colon, lung, breast, up-regulated Iorio et al., 2005 [4]

miR-21 stomach, prostate Volinia et al., 2006 [5]

miR-142-5p, miR-369-3p, lung cancer up-regulated Baffa et al., 2009 [6]

miR-215

miR-373 lung cancer down-regulated Baffa et al., 2009 [6]

miR-30d, miR-125b,

miR-26a, miR-30a-5p thyroid anaplastic down-regulated Visone et al., 2007 [7]

carcinomas

miR-10b, miR-125b, breast cancer down-regulated Iorio et al., 2005 [4]

miR-145

miR-27a, miR-96, breast cancer up-regulated Gutilla et al., 2009 [8]

miR-182

miR-21, miR-155 breast cancer up-regulated Iorio et al., 2005 [4]

miR-21 breast cancer up-regulated Huang et al., 2009 [9]

miR-30b, miR148a breast cancer up-regulated Baffa et al., 2009 [6]

miR-205 breast cancer down-regulated Baffa et al., 2009 [6]

miR-142-5p, miR-29b, bladder cancer up-regulated Baffa et al., 2009 [6]

miR-30b

miR-145, miR-143, miR-320 bladder cancer down-regulated Baffa et al., 2009 [6]

miR-138, miR-125b colon cancer up-regulated Baffa et al., 2009 [6]

miR-17, miR-106a colon cancer down-regulated Baffa et al., 2009 [6]

hsa-miR-205 head and neck cancer up-regulated Tran et al., 2007 [10]

miR-21, miR-221 brain cancer up-regulated Ciafre et al., 2005 [11]

miR-9-2, miR-10b glioblastomas up-regulated Ciafre et al., 2005 [11]

miR-21, miR-25, miR-123

miR-125b-1, miR-125b-2

miR-130a, miR-221

miR-10b, miR-21, miR-26a glioblastomas up-regulated Godlewski et al., 2008

miR-383, miR-451, [12]

miR-486, miR-516-3p,

miR-519d

miR-10b, miR-21, glioblastomas up-regulated Silber et al., 2008 [13]

miR-155, miR-210

miR-16, miR-107, glioblastomas up-regulated Gal et al., 2008 [14]

miR-185, miR-425,

miR-451, miR-486

miR-128-1, miR-181a glioblastomas down-regulated Ciafre et al., 2005 [11]

miR-181b, miR-181c

miR-124, miR-128-1, glioblastomas down-regulated Godlewski et al., 2008

miR-128-2, miR-137, [12]

miR-139, miR-190,

miR-218, miR-299,

miR-323, miR-483,

miR-511-1

miR-7, miR-29b, miR-31 glioblastomas down-regulated Silber et al., 2008 [13]

miR-101, miR-107,

miR-124, miR-124-2,

miR-128-1, miR-129,

miR-132, miR-133a ,

miR-133b, miR-137

miR-138, miR-139, glioblastomas down-regulated Gal et al., 2008 [14]

miR-149, miR-153,

miR-154, miR-185,

miR-187, miR-203,

miR-218, miR-323,

miR-328, miR-330

miR-15, miR-16 CLL down-regulated Calin et al., 2002 [15]

miR-150, miR-155 CLL up-regulated Bartels et al., 2009 [16]

miR-143, miR-145 colorectal neoplasia down-regulated Michael et al., 2003

[17]

miR-18, miR-224 hepato. Carcinoma up-regulated Murakami et al., 2006

[18]

miR-199, miR-195, hepato. Carcinoma down-regulated Murakami et al., 2006

miR-200, miR-125 [18]

miR-155, miR-17–92 lymphomas up-regulated Eis et al., 2005 [19]

miR-221, miR-222, papillary thyroid

miR-146, miR-181 carcinoma up-regulated He et al., 2005b; [20]

Pallante et al., 2006[21]

miR-372, miR-373 testicular germ cell

tumors up-regulated Voorhoeve et al., 2006

[22]

miR-31, miR-96, miR-135b, colorectal cancer up-regulated Bartels et al., 2009 [16]

miR-183

miR-48, miR-135b colorectal cancer down-regulated Bartels et al., 2009 [16]

miR-133b Bandres et al., 2006[23]

let-7b, let-7 g, miR-9, colorectal cancer up-regulated Yang et al., 2009 [24]

miR-21, miR-26a, miR-30a-3p,

miR-30a-5p, miR-31, miR-96,

miR-124b, miR-132, miR-135a,

miR-135b, miR-141,

miR-142-3p, miR-142-5p,

miR-181a, miR-181b,

miR-182, miR-183, miR-194,

miR-200a, miR-200b,

miR-200c, miR-203,

miR-205, miR-215, miR-219,

miR-320, miR-338, miR-372

let-7a, miR-10a, colorectal cancer down-regulated Yang et al., 2009 [24]

miR-15b, miR-23a, miR-25,

miR-27a, miR-27b, miR-30c,

miR-107, miR-124a,

miR0125a, miR-125b, miR-127,

miR-130a, miR-133a, miR-133b,

miR-134, miR-137, miR-143,

miR-145, miR-147, miR-154,

miR-191, miR-199a, miR-199b,

miR-214, miR-296, miR-299,

miR-337, miR-339, miR-342,

miR-368, miR-370, miR-582

miR-224, miR-18 and Hepatocellular cancer up-regulated Murakami et al., 2006

[18]

pre-miR-P18

miR-221 Hepatocellular cancer up-regulated Fornari et al., 2008 [25]

miR-199a, miR-199a*, Hepatocellular cancer down-regulated Murakami et al., 2006

miR-200a, miR-125a, [18]

miR-195

miR-125b Hepatocellular cancer down-regulated Li et al., 2008 [26]

**References for additional file 1**

1. Porkka KP, Pfeiffer MJ, Waltering KK, Vessella RL, Tammela TL, Visakorpi T: [**MicroRNA expression profiling in prostate cancer.**](http://www.ncbi.nlm.nih.gov/pubmed/17616669?ordinalpos=1&itool=EntrezSystem2.PEntrez.Pubmed.Pubmed_ResultsPanel.Pubmed_DefaultReportPanel.Pubmed_RVDocSum)Cancer Res 2007, Jul 1; **67(13):**6130-5.
2. Lodes MJ, Caraballo M, Suciu D, Munro S, Kumar A, Anderson B: [**Detection of cancer with serum miRNAs on an oligonucleotide microarray.**](http://www.ncbi.nlm.nih.gov/pubmed/19597549?ordinalpos=1&itool=EntrezSystem2.PEntrez.Pubmed.Pubmed_ResultsPanel.Pubmed_DefaultReportPanel.Pubmed_RVDocSum) PLoS One 2009, Jul 14; **4 (7):**e6229.
3. Bloomston M, Frankel WL, Petrocca F, Volinia S, Alder H, Hagan JP, Liu CG, Bhatt D, Taccioli C, Croce CM: **MicroRNA expression patterns to differentiate pancreatic adenocarcinoma from normal pancreas and chronic pancreatitis** JAMA 2007, **297(17):**1901-1908 (doi:10.1001/jama.297.17.1901).
4. Iorio MV, Ferracin M, Liu CG, Veronese A, Spizzo R, Sabbioni S, Magri A, Musiani P, Volinia S, Nenci I, Calin GA, Querzzoli P: **MicroRNA gene expression deregulation in human breast cancer.** Cancer Res 2005, **65:**7065–7070.
5. Volinia S, Calin GA, Liu CG, Ambs S, Cimmino A, Petrocca F, Visone R, Iorio M, Roldo C, Ferracin M, Prueitt RL, Yanaihara N, Lanza G, Scarpa A, Vecchione A, Negrini M, Harris CC, Croce CM: [**A microRNA expression signature of human solid tumors defines cancer gene targets.**](http://www.ncbi.nlm.nih.gov/pubmed/16461460?ordinalpos=11&itool=EntrezSystem2.PEntrez.Pubmed.Pubmed_ResultsPanel.Pubmed_DefaultReportPanel.Pubmed_RVDocSum) Proc Natl Acad Sci USA 2006, Feb 14; **103(7):**2257-2261. Epub 2006 Feb 3.
6. [Baffa R](http://www.ncbi.nlm.nih.gov/sites/entrez?Db=pubmed&Cmd=Search&Term="Baffa R"%5BAuthor%5D&itool=EntrezSystem2.PEntrez.Pubmed.Pubmed_ResultsPanel.Pubmed_DiscoveryPanel.Pubmed_RVAbstractPlus), [Fassan M](http://www.ncbi.nlm.nih.gov/sites/entrez?Db=pubmed&Cmd=Search&Term="Fassan M"%5BAuthor%5D&itool=EntrezSystem2.PEntrez.Pubmed.Pubmed_ResultsPanel.Pubmed_DiscoveryPanel.Pubmed_RVAbstractPlus), [Volinia S](http://www.ncbi.nlm.nih.gov/sites/entrez?Db=pubmed&Cmd=Search&Term="Volinia S"%5BAuthor%5D&itool=EntrezSystem2.PEntrez.Pubmed.Pubmed_ResultsPanel.Pubmed_DiscoveryPanel.Pubmed_RVAbstractPlus), [O'Hara B](http://www.ncbi.nlm.nih.gov/sites/entrez?Db=pubmed&Cmd=Search&Term="O'Hara B"%5BAuthor%5D&itool=EntrezSystem2.PEntrez.Pubmed.Pubmed_ResultsPanel.Pubmed_DiscoveryPanel.Pubmed_RVAbstractPlus), [Liu CG](http://www.ncbi.nlm.nih.gov/sites/entrez?Db=pubmed&Cmd=Search&Term="Liu CG"%5BAuthor%5D&itool=EntrezSystem2.PEntrez.Pubmed.Pubmed_ResultsPanel.Pubmed_DiscoveryPanel.Pubmed_RVAbstractPlus), [Palazzo JP](http://www.ncbi.nlm.nih.gov/sites/entrez?Db=pubmed&Cmd=Search&Term="Palazzo JP"%5BAuthor%5D&itool=EntrezSystem2.PEntrez.Pubmed.Pubmed_ResultsPanel.Pubmed_DiscoveryPanel.Pubmed_RVAbstractPlus), [Gardiman M](http://www.ncbi.nlm.nih.gov/sites/entrez?Db=pubmed&Cmd=Search&Term="Gardiman M"%5BAuthor%5D&itool=EntrezSystem2.PEntrez.Pubmed.Pubmed_ResultsPanel.Pubmed_DiscoveryPanel.Pubmed_RVAbstractPlus), [Rugge M](http://www.ncbi.nlm.nih.gov/sites/entrez?Db=pubmed&Cmd=Search&Term="Rugge M"%5BAuthor%5D&itool=EntrezSystem2.PEntrez.Pubmed.Pubmed_ResultsPanel.Pubmed_DiscoveryPanel.Pubmed_RVAbstractPlus), [Gomella LG](http://www.ncbi.nlm.nih.gov/sites/entrez?Db=pubmed&Cmd=Search&Term="Gomella LG"%5BAuthor%5D&itool=EntrezSystem2.PEntrez.Pubmed.Pubmed_ResultsPanel.Pubmed_DiscoveryPanel.Pubmed_RVAbstractPlus), [Croce CM](http://www.ncbi.nlm.nih.gov/sites/entrez?Db=pubmed&Cmd=Search&Term="Croce CM"%5BAuthor%5D&itool=EntrezSystem2.PEntrez.Pubmed.Pubmed_ResultsPanel.Pubmed_DiscoveryPanel.Pubmed_RVAbstractPlus), [Rosenberg A](http://www.ncbi.nlm.nih.gov/sites/entrez?Db=pubmed&Cmd=Search&Term="Rosenberg A"%5BAuthor%5D&itool=EntrezSystem2.PEntrez.Pubmed.Pubmed_ResultsPanel.Pubmed_DiscoveryPanel.Pubmed_RVAbstractPlus): **MicroRNA expression profiling of human metastatic cancers identifies cancer gene targets.** [J Pathol](javascript:AL_get(this, 'jour', 'J Pathol.');) 2009, Jun 1. [Epub ahead of print].
7. [Visone R](http://www.ncbi.nlm.nih.gov/sites/entrez?Db=pubmed&Cmd=Search&Term="Visone R"%5BAuthor%5D&itool=EntrezSystem2.PEntrez.Pubmed.Pubmed_ResultsPanel.Pubmed_DiscoveryPanel.Pubmed_RVAbstractPlus), [Pallante P](http://www.ncbi.nlm.nih.gov/sites/entrez?Db=pubmed&Cmd=Search&Term="Pallante P"%5BAuthor%5D&itool=EntrezSystem2.PEntrez.Pubmed.Pubmed_ResultsPanel.Pubmed_DiscoveryPanel.Pubmed_RVAbstractPlus), [Vecchione A](http://www.ncbi.nlm.nih.gov/sites/entrez?Db=pubmed&Cmd=Search&Term="Vecchione A"%5BAuthor%5D&itool=EntrezSystem2.PEntrez.Pubmed.Pubmed_ResultsPanel.Pubmed_DiscoveryPanel.Pubmed_RVAbstractPlus), [Cirombella R](http://www.ncbi.nlm.nih.gov/sites/entrez?Db=pubmed&Cmd=Search&Term="Cirombella R"%5BAuthor%5D&itool=EntrezSystem2.PEntrez.Pubmed.Pubmed_ResultsPanel.Pubmed_DiscoveryPanel.Pubmed_RVAbstractPlus), [Ferracin M](http://www.ncbi.nlm.nih.gov/sites/entrez?Db=pubmed&Cmd=Search&Term="Ferracin M"%5BAuthor%5D&itool=EntrezSystem2.PEntrez.Pubmed.Pubmed_ResultsPanel.Pubmed_DiscoveryPanel.Pubmed_RVAbstractPlus), [Ferraro A](http://www.ncbi.nlm.nih.gov/sites/entrez?Db=pubmed&Cmd=Search&Term="Ferraro A"%5BAuthor%5D&itool=EntrezSystem2.PEntrez.Pubmed.Pubmed_ResultsPanel.Pubmed_DiscoveryPanel.Pubmed_RVAbstractPlus), [Volinia S](http://www.ncbi.nlm.nih.gov/sites/entrez?Db=pubmed&Cmd=Search&Term="Volinia S"%5BAuthor%5D&itool=EntrezSystem2.PEntrez.Pubmed.Pubmed_ResultsPanel.Pubmed_DiscoveryPanel.Pubmed_RVAbstractPlus), [Coluzzi S](http://www.ncbi.nlm.nih.gov/sites/entrez?Db=pubmed&Cmd=Search&Term="Coluzzi S"%5BAuthor%5D&itool=EntrezSystem2.PEntrez.Pubmed.Pubmed_ResultsPanel.Pubmed_DiscoveryPanel.Pubmed_RVAbstractPlus), [Leone V](http://www.ncbi.nlm.nih.gov/sites/entrez?Db=pubmed&Cmd=Search&Term="Leone V"%5BAuthor%5D&itool=EntrezSystem2.PEntrez.Pubmed.Pubmed_ResultsPanel.Pubmed_DiscoveryPanel.Pubmed_RVAbstractPlus), [Borbone E](http://www.ncbi.nlm.nih.gov/sites/entrez?Db=pubmed&Cmd=Search&Term="Borbone E"%5BAuthor%5D&itool=EntrezSystem2.PEntrez.Pubmed.Pubmed_ResultsPanel.Pubmed_DiscoveryPanel.Pubmed_RVAbstractPlus), [Liu CG](http://www.ncbi.nlm.nih.gov/sites/entrez?Db=pubmed&Cmd=Search&Term="Liu CG"%5BAuthor%5D&itool=EntrezSystem2.PEntrez.Pubmed.Pubmed_ResultsPanel.Pubmed_DiscoveryPanel.Pubmed_RVAbstractPlus), [Petrocca F](http://www.ncbi.nlm.nih.gov/sites/entrez?Db=pubmed&Cmd=Search&Term="Petrocca F"%5BAuthor%5D&itool=EntrezSystem2.PEntrez.Pubmed.Pubmed_ResultsPanel.Pubmed_DiscoveryPanel.Pubmed_RVAbstractPlus), [Troncone G](http://www.ncbi.nlm.nih.gov/sites/entrez?Db=pubmed&Cmd=Search&Term="Troncone G"%5BAuthor%5D&itool=EntrezSystem2.PEntrez.Pubmed.Pubmed_ResultsPanel.Pubmed_DiscoveryPanel.Pubmed_RVAbstractPlus), [Calin GA](http://www.ncbi.nlm.nih.gov/sites/entrez?Db=pubmed&Cmd=Search&Term="Calin GA"%5BAuthor%5D&itool=EntrezSystem2.PEntrez.Pubmed.Pubmed_ResultsPanel.Pubmed_DiscoveryPanel.Pubmed_RVAbstractPlus), [Scarpa A](http://www.ncbi.nlm.nih.gov/sites/entrez?Db=pubmed&Cmd=Search&Term="Scarpa A"%5BAuthor%5D&itool=EntrezSystem2.PEntrez.Pubmed.Pubmed_ResultsPanel.Pubmed_DiscoveryPanel.Pubmed_RVAbstractPlus), [Colato C](http://www.ncbi.nlm.nih.gov/sites/entrez?Db=pubmed&Cmd=Search&Term="Colato C"%5BAuthor%5D&itool=EntrezSystem2.PEntrez.Pubmed.Pubmed_ResultsPanel.Pubmed_DiscoveryPanel.Pubmed_RVAbstractPlus), [Tallini G](http://www.ncbi.nlm.nih.gov/sites/entrez?Db=pubmed&Cmd=Search&Term="Tallini G"%5BAuthor%5D&itool=EntrezSystem2.PEntrez.Pubmed.Pubmed_ResultsPanel.Pubmed_DiscoveryPanel.Pubmed_RVAbstractPlus), [Santoro M](http://www.ncbi.nlm.nih.gov/sites/entrez?Db=pubmed&Cmd=Search&Term="Santoro M"%5BAuthor%5D&itool=EntrezSystem2.PEntrez.Pubmed.Pubmed_ResultsPanel.Pubmed_DiscoveryPanel.Pubmed_RVAbstractPlus), [Croce CM](http://www.ncbi.nlm.nih.gov/sites/entrez?Db=pubmed&Cmd=Search&Term="Croce CM"%5BAuthor%5D&itool=EntrezSystem2.PEntrez.Pubmed.Pubmed_ResultsPanel.Pubmed_DiscoveryPanel.Pubmed_RVAbstractPlus), [Fusco A](http://www.ncbi.nlm.nih.gov/sites/entrez?Db=pubmed&Cmd=Search&Term="Fusco A"%5BAuthor%5D&itool=EntrezSystem2.PEntrez.Pubmed.Pubmed_ResultsPanel.Pubmed_DiscoveryPanel.Pubmed_RVAbstractPlus): **Specific microRNAs are down regulated in human thyroid anaplastic carcinomas.** [Oncogene](javascript:AL_get(this, 'jour', 'Oncogene.');) 2007, Nov 29; **26(54):**7590-7595. Epub 2007 Jun 11.
8. Guttilla IK, White BA: [**Coordinate regulation of FOXO1 by miR-27a, miR-96, and miR-182 in breast cancer cells.**](http://www.ncbi.nlm.nih.gov/pubmed/19574223?ordinalpos=2&itool=EntrezSystem2.PEntrez.Pubmed.Pubmed_ResultsPanel.Pubmed_DefaultReportPanel.Pubmed_RVDocSum) J Biol Chem 2009 Jul 1. [Epub ahead of print].
9. Huang GL, Zhang XH, Guo GL, Huang KT, Yang KY, Shen X, You J, Hu XQ: [**Clinical significance of miR-21 expression in breast cancer: SYBR-Green I-based real-time RT-PCR study of invasive ductal carcinoma.**](http://www.ncbi.nlm.nih.gov/pubmed/19212625?ordinalpos=4&itool=EntrezSystem2.PEntrez.Pubmed.Pubmed_ResultsPanel.Pubmed_DefaultReportPanel.Pubmed_RVDocSum)Oncol Rep. 2009, Mar 21; **(3):**673-9.
10. Tran N, McLean T, Zhang X, Zhao CJ, Thomson JM, O’Brien C, Rose B: **MicroRNA expression profiles in head and neck cancer cell lines.** Biochem Biophys Res Commun 2007, **358:**12–17.
11. Ciafre, S.A., Galardi, S., Mangiola, A., Ferracin, M., Liu, C.G., Sabatino, G., Negrini, M., Maira, G., Croce, C.M., Farace, and M.G: **Extensive modulation of a set of microRNAs in primary glioblastomas.** Biochem Biophys Res Commun 2005, **334:**1351–1358.
12. Godlewski J, Nowicki MO, Bronisz A, Williams S, Otsuki A, Nuovo G, Raychaudhury A, Newton HB, Chiocca EA, Lawler S: **Targeting of the Bmi-1 oncogene/stem cell renewal factor by microRNA-128 inhibits glioma proliferation and self-renewal.** Cancer Res 2008, **68:**9125–9130. doi:10.1158/0008-5472.CAN-08-2629.
13. Silber J, Lim DA, Petritsch C, Persson AI, Maunakea AK, Yu M, Vandenberg SR, Ginzinger DG, James CD, Costello JF, Bergers G, Weiss WA, Alvarez-Buylla A, Hodgson JG: **miR-124 and miR-137 inhibit proliferation of glioblastoma multiform cells and induce differentiation of brain tumor stem cells.** BMC Med 2008, **6:**14. doi:10.1186/1741-7015-6-14.
14. Gal H, Pandi G, Kanner AA, Ram Z, Lithwick-Yanai G, Amariglio N, Rechavi G, Givol D: **MIR-451 and Imatinib mesylate inhibit tumor growth of glioblastoma stem cells.** Biochem Biophys Res Commun 2008, **376:**86–90. doi:10.1016/j.bbrc. 2008.08.107.
15. Calin GA: **Frequent deletions and down-regulation of micro-RNA genes miR15 and miR16 at 13q14 in chronic lymphocytic leukemia**. Proc Natl Acad Sci USA 2002, **99:** 15524–15529.
16. [Bartels CL](http://www.ncbi.nlm.nih.gov/sites/entrez?Db=pubmed&Cmd=Search&Term="Bartels CL"%5BAuthor%5D&itool=EntrezSystem2.PEntrez.Pubmed.Pubmed_ResultsPanel.Pubmed_DiscoveryPanel.Pubmed_RVAbstractPlus), [Tsongalis GJ](http://www.ncbi.nlm.nih.gov/sites/entrez?Db=pubmed&Cmd=Search&Term="Tsongalis GJ"%5BAuthor%5D&itool=EntrezSystem2.PEntrez.Pubmed.Pubmed_ResultsPanel.Pubmed_DiscoveryPanel.Pubmed_RVAbstractPlus): **MicroRNAs: novel biomarkers for human cancer.** [Clin Chem](javascript:AL_get(this, 'jour', 'Clin Chem.');) 2009, Apr; **55(4):**623-31. Epub 2009 Feb 26.
17. Michael MZ, O´Connor SM, Van Holst Pellekaan NG, Young GP, James RJ: **Reduced accumulation of specific microRNAs in colorectal neoplasia.** Mol Cancer Res 2003, **1:**882-891.
18. Murakami Y, Yasuda T, Saigo K, Urashima T, Toyoda H, Okanoue T, Shimotohno K: **Comprehensive analysis of microRNA expression patterns in hepatocellular carcinoma and non-timorous tissues.** Oncogene 2006, **25:**2537–2545.
19. Eis PS, Tam W, Sun LP, Chadburn A, Li Z.D, Gomez MF, Lund E, Dahlberg JE: **Accumulation of miR-155 and BIC RNA in human B cell lymphomas.** Proc Natl Acad Sci USA 2005, **102:** 3627–3632. Epub 2005 Feb 28.
20. He H, Jazdzewski K, Li W, Liyanarachchi S, Nagi R, Volinia S, Calin GA: **The role of microRNA genes in papillary thyroid carcinoma.** Proc Natl Acad Sci USA 2005b, **102:**19075-19080.
21. Pallante P, Visone R, Ferracin M, Ferraro A, Berlingieri MT, Troncone G, Chiappetta G, Liu CG, Santoro M, Negrini M: **Deregulation in human thyroid papillary carcinomas.** Endocr Relat Cancer 2006, **13:**497–508.
22. Voorhoeve PM, le Sage C, Schrier M: **Genetic screen implicates miRNA-372 and miRNA-373 as oncogenes in testicular germ cell tumors.** Cell 2006, **124:**1169-1181.
23. Bandres E, Cubedo E, Agirre X, Malumbres R, Zarate R, Ramirez N, Abajo A: **Identification by Real-time PCR of 13 mature microRNAs differentially expressed in colorectal cancer and nontumoral tissues.** Mol Cancer 2006, **5:**29.
24. Yang L, Belaguli N, Berger DH: **MicroRNA and Colorectal Cancer.** World J Surg 2009, **33:**638–646.
25. Fornari F, Gramantieri L, Ferracin M, Veronese A, Sabbioni S, Calin GA, Grazi GL, Giovannini C, Croce CM, Bolondi L, Negrini M: [**MiR-221 controls CDKN1C/p57 and CDKN1B/p27 expression in human hepatocellular carcinoma.**](http://www.ncbi.nlm.nih.gov/pubmed/18521080?ordinalpos=2&itool=EntrezSystem2.PEntrez.Pubmed.Pubmed_ResultsPanel.Pubmed_DefaultReportPanel.Pubmed_RVDocSum) Oncogene 2008, Sep 25; **27(43):**5651-61 Epub 2008 Jun 2.
26. [Li W](http://www.ncbi.nlm.nih.gov/sites/entrez?Db=pubmed&Cmd=Search&Term="Li W"%5BAuthor%5D&itool=EntrezSystem2.PEntrez.Pubmed.Pubmed_ResultsPanel.Pubmed_DiscoveryPanel.Pubmed_RVAbstractPlus), [Xie L](http://www.ncbi.nlm.nih.gov/sites/entrez?Db=pubmed&Cmd=Search&Term="Xie L"%5BAuthor%5D&itool=EntrezSystem2.PEntrez.Pubmed.Pubmed_ResultsPanel.Pubmed_DiscoveryPanel.Pubmed_RVAbstractPlus), [He X](http://www.ncbi.nlm.nih.gov/sites/entrez?Db=pubmed&Cmd=Search&Term="He X"%5BAuthor%5D&itool=EntrezSystem2.PEntrez.Pubmed.Pubmed_ResultsPanel.Pubmed_DiscoveryPanel.Pubmed_RVAbstractPlus), [Li J](http://www.ncbi.nlm.nih.gov/sites/entrez?Db=pubmed&Cmd=Search&Term="Li J"%5BAuthor%5D&itool=EntrezSystem2.PEntrez.Pubmed.Pubmed_ResultsPanel.Pubmed_DiscoveryPanel.Pubmed_RVAbstractPlus), [Tu K](http://www.ncbi.nlm.nih.gov/sites/entrez?Db=pubmed&Cmd=Search&Term="Tu K"%5BAuthor%5D&itool=EntrezSystem2.PEntrez.Pubmed.Pubmed_ResultsPanel.Pubmed_DiscoveryPanel.Pubmed_RVAbstractPlus), [Wei L](http://www.ncbi.nlm.nih.gov/sites/entrez?Db=pubmed&Cmd=Search&Term="Wei L"%5BAuthor%5D&itool=EntrezSystem2.PEntrez.Pubmed.Pubmed_ResultsPanel.Pubmed_DiscoveryPanel.Pubmed_RVAbstractPlus), [Wu J](http://www.ncbi.nlm.nih.gov/sites/entrez?Db=pubmed&Cmd=Search&Term="Wu J"%5BAuthor%5D&itool=EntrezSystem2.PEntrez.Pubmed.Pubmed_ResultsPanel.Pubmed_DiscoveryPanel.Pubmed_RVAbstractPlus), [Guo Y](http://www.ncbi.nlm.nih.gov/sites/entrez?Db=pubmed&Cmd=Search&Term="Guo Y"%5BAuthor%5D&itool=EntrezSystem2.PEntrez.Pubmed.Pubmed_ResultsPanel.Pubmed_DiscoveryPanel.Pubmed_RVAbstractPlus), [Ma X](http://www.ncbi.nlm.nih.gov/sites/entrez?Db=pubmed&Cmd=Search&Term="Ma X"%5BAuthor%5D&itool=EntrezSystem2.PEntrez.Pubmed.Pubmed_ResultsPanel.Pubmed_DiscoveryPanel.Pubmed_RVAbstractPlus), [Zhang P](http://www.ncbi.nlm.nih.gov/sites/entrez?Db=pubmed&Cmd=Search&Term="Zhang P"%5BAuthor%5D&itool=EntrezSystem2.PEntrez.Pubmed.Pubmed_ResultsPanel.Pubmed_DiscoveryPanel.Pubmed_RVAbstractPlus), [Pan Z](http://www.ncbi.nlm.nih.gov/sites/entrez?Db=pubmed&Cmd=Search&Term="Pan Z"%5BAuthor%5D&itool=EntrezSystem2.PEntrez.Pubmed.Pubmed_ResultsPanel.Pubmed_DiscoveryPanel.Pubmed_RVAbstractPlus), [Hu X](http://www.ncbi.nlm.nih.gov/sites/entrez?Db=pubmed&Cmd=Search&Term="Hu X"%5BAuthor%5D&itool=EntrezSystem2.PEntrez.Pubmed.Pubmed_ResultsPanel.Pubmed_DiscoveryPanel.Pubmed_RVAbstractPlus), [Zhao Y](http://www.ncbi.nlm.nih.gov/sites/entrez?Db=pubmed&Cmd=Search&Term="Zhao Y"%5BAuthor%5D&itool=EntrezSystem2.PEntrez.Pubmed.Pubmed_ResultsPanel.Pubmed_DiscoveryPanel.Pubmed_RVAbstractPlus), [Xie H](http://www.ncbi.nlm.nih.gov/sites/entrez?Db=pubmed&Cmd=Search&Term="Xie H"%5BAuthor%5D&itool=EntrezSystem2.PEntrez.Pubmed.Pubmed_ResultsPanel.Pubmed_DiscoveryPanel.Pubmed_RVAbstractPlus), [Jiang G](http://www.ncbi.nlm.nih.gov/sites/entrez?Db=pubmed&Cmd=Search&Term="Jiang G"%5BAuthor%5D&itool=EntrezSystem2.PEntrez.Pubmed.Pubmed_ResultsPanel.Pubmed_DiscoveryPanel.Pubmed_RVAbstractPlus), [Chen T](http://www.ncbi.nlm.nih.gov/sites/entrez?Db=pubmed&Cmd=Search&Term="Chen T"%5BAuthor%5D&itool=EntrezSystem2.PEntrez.Pubmed.Pubmed_ResultsPanel.Pubmed_DiscoveryPanel.Pubmed_RVAbstractPlus), [Wang J](http://www.ncbi.nlm.nih.gov/sites/entrez?Db=pubmed&Cmd=Search&Term="Wang J"%5BAuthor%5D&itool=EntrezSystem2.PEntrez.Pubmed.Pubmed_ResultsPanel.Pubmed_DiscoveryPanel.Pubmed_RVAbstractPlus), [Zheng S](http://www.ncbi.nlm.nih.gov/sites/entrez?Db=pubmed&Cmd=Search&Term="Zheng S"%5BAuthor%5D&itool=EntrezSystem2.PEntrez.Pubmed.Pubmed_ResultsPanel.Pubmed_DiscoveryPanel.Pubmed_RVAbstractPlus), [Cheng J](http://www.ncbi.nlm.nih.gov/sites/entrez?Db=pubmed&Cmd=Search&Term="Cheng J"%5BAuthor%5D&itool=EntrezSystem2.PEntrez.Pubmed.Pubmed_ResultsPanel.Pubmed_DiscoveryPanel.Pubmed_RVAbstractPlus), [Wan D](http://www.ncbi.nlm.nih.gov/sites/entrez?Db=pubmed&Cmd=Search&Term="Wan D"%5BAuthor%5D&itool=EntrezSystem2.PEntrez.Pubmed.Pubmed_ResultsPanel.Pubmed_DiscoveryPanel.Pubmed_RVAbstractPlus), [Yang S](http://www.ncbi.nlm.nih.gov/sites/entrez?Db=pubmed&Cmd=Search&Term="Yang S"%5BAuthor%5D&itool=EntrezSystem2.PEntrez.Pubmed.Pubmed_ResultsPanel.Pubmed_DiscoveryPanel.Pubmed_RVAbstractPlus), [Li Y](http://www.ncbi.nlm.nih.gov/sites/entrez?Db=pubmed&Cmd=Search&Term="Li Y"%5BAuthor%5D&itool=EntrezSystem2.PEntrez.Pubmed.Pubmed_ResultsPanel.Pubmed_DiscoveryPanel.Pubmed_RVAbstractPlus), [Gu J](http://www.ncbi.nlm.nih.gov/sites/entrez?Db=pubmed&Cmd=Search&Term="Gu J"%5BAuthor%5D&itool=EntrezSystem2.PEntrez.Pubmed.Pubmed_ResultsPanel.Pubmed_DiscoveryPanel.Pubmed_RVAbstractPlus): **Diagnostic and prognostic implications of microRNAs in human hepatocellular carcinoma.** [Int J Cancer](javascript:AL_get(this, 'jour', 'Int J Cancer.');) 2008, Oct 1; **123(7):**1616-22.
